# Effect of bacterial resistant zwitterionic derivative incorporation on the physical properties of resin-modified glass ionomer luting cement

**DOI:** 10.1038/s41598-023-30670-4

**Published:** 2023-03-03

**Authors:** Chengzan Wu, Min-Ji Kim, Utkarsh Mangal, Ji-Young Seo, Ji-Yeong Kim, Junho Kim, Ju-Young Park, Jae-Sung Kwon, Sung-Hwan Choi

**Affiliations:** 1 Department of Orthodontics and Institute of Craniofacial Deformity, Yonsei University College of Dentistry, 50-1 Yonsei-Ro, Seodaemun-Gu, Seoul, 03722 Republic of Korea; 2 Division in Anatomy and Developmental Biology, Department of Oral Biology, Yonsei University College of Dentistry, 50-1 Yonsei-Ro, Seodaemun-Gu, Seoul, 03722 Republic of Korea; 3 Institute of Craniofacial Deformity, Yonsei University College of Dentistry, 50-1 Yonsei-Ro, Seodaemun-Gu, Seoul, 03722 Republic of Korea; 4 BK21 FOUR Project, Yonsei University College of Dentistry, 50-1 Yonsei-Ro, Seodaemun-Gu, Seoul, 03722 Republic of Korea; 5 Department and Research Institute of Dental Biomaterials and Bioengineering, BK21 PLUS Project, Yonsei University College of Dentistry, 50-1 Yonsei-Ro, Seodaemun-Gu, Seoul, 03722 Republic of Korea

**Keywords:** Dental materials, Glass-ionomer cement

## Abstract

Biofilms induce microbial-mediated surface roughening and deterioration of cement. In this study, zwitterionic derivatives (ZD) of sulfobetaine methacrylate (SBMA) and 2-methacryloyloxyethyl phosphorylcholine, were added in concentrations of 0, 1, and 3% to three different types of commercially available resin-modified glass ionomer cement (RMGIC) (RMC-I: RelyX Luting 2, RMC-II: Nexus RMGI, and RMC-III: GC FujiCEM 2). The unmodified RMGICs served as the control group for comparison. The resistance of *Streptococcus mutans* to ZD-modified RMGIC was evaluated with a monoculture biofilm assay. The following physical properties of the ZD-modified RMGIC were assessed: wettability, film thickness, flexural strength, elastic modulus, shear bond strength, and failure mode. The ZD-modified RMGIC significantly inhibited biofilm formation, with at least a 30% reduction compared to the control group. The addition of ZD improved the wettability of RMGIC; however, only 3% of the SBMA group was statistically different (*P* < 0.05). The film thickness increased in proportion to the increasing ZD concentrations; there was no statistical difference within the RMC-I (*P* > 0.05). The experimental groups' flexural strength, elastic modulus, and shear bond strength showed an insignificant decrease from the control group; there was no statistical difference within the RMC-I (*P* > 0.05). The mode of failure differed slightly in each group, but all groups showed dominance in the adhesive and mixed failure. Thus, the addition of 1 wt.% ZD in RMGIC favorably enhanced the resistance to *Streptococcus mutans* without any tangible loss in flexural and shear bond strength.

## Introduction

In recent years, all-ceramic restorations such as zirconia have widely replaced metal, and other porcelain mixed restorations in dental practice. The latest metal-free materials offer supreme properties in color stability, translucency, and light transmission to resemble the natural tooth and, as a result, meet optimal patient esthetic expectations^1^. As zirconia is highly considered the material of choice for indirect all-ceramic restorations, cementing zirconia has become increasingly important. Therefore, for long-term clinical success of fixed prosthodontic restorations, an appropriate bonding procedure is mandatory where the luting agent plays an essential role in esthetics and function^2^.

Dental luting cement generally provides mechanical lock by filling the tiny gap between the tooth (or implant abutment) and the restoration, which subsequently maintains integration of the two surfaces and prevents dislodgement of the restoration during function^3,4^. In this regard, an ideal luting cement must satisfy specific biological, physical, and mechanical requirements. Among the current luting agents, resin-modified glass ionomer cement (RMGIC) is a suitable choice for the cementation of zirconia ceramic restorations^5^.

A widely used clinical bonding material, RMGIC was developed by incorporating resin components into a conventional glass ionomer cement (GIC)^6^. The main advantage of RMGIC includes fluoride ion release, bonding to enamel and dentin, relative ease of use, and acceptable long-term esthetic quality^7^. The esthetic and biological compatibilities of RMGIC have made it preferable for bonding esthetic crowns. RMGIC provides a mechanical bond to the restoration, and a limited yet effective chemical bond to the underlying tooth structure, thus forming a crucial interface^8^.

However, the cement interface is continuously targeted by various challenges; microleakage is a common and persistent issue. Fixed prosthetic restorations with marginal gaps run the risk of microleakage, biofilm accumulation, and tooth demineralization, resulting from the tooth-cement interface's exposure to the oral cavity and subsequent collection of food debris in the exposed gap^9,10^.

**Figure 1:**
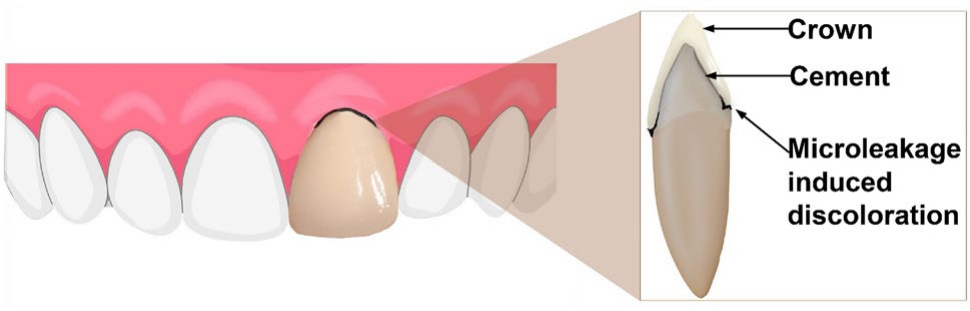
Schematic representation of luting cement failure leading to discoloration, loss of esthetics, and secondary carious lesions.

Moreover, compared to gold and amalgam, that are predisposed to thick and largely non-viable biofilm, ceramic surfaces acquire thin and highly viable biofilm leading to a greater tendency of bacterial biofilm formation^11^. Biofilm induces microbial-mediated surface roughening and deterioration of cement^12^. This leads to a chronic biofilm formation at the cement interface, leading to restoration failure with or without secondary caries^13,14^ (Fig. 1).

Alternative strategies to combat biofilm-induced damage is an active topic discussed in biomaterial and clinical research^15^. In this respect, a repellent tactic has been developed to prevent pellicles from attaching to the material surface, thereby preventing the primary colonization of bacteria and breaking the biofilm cycle^15,16^. Protein-repellent composites can reduce bacteria adhesion, thus decreasing biofilm growth on the material surface while minimizing the local and systemic adverse effects^17^.

A promising approach in dental biomaterial modification has been observed in using zwitterionic derivatives (ZD). ZD are substances with strong hydration properties embracing both cationic and anionic groups, which can effectively resist non-specific protein adsorption by bound water mechanism^18–20^. Polybetaine ZD, such as the sulfobetaine methacrylate (SBMA) and 2-Methacryloyloxyethyl phosphorylcholine (MPC) is commonly used for its biocompatibility with oral tissues and ease of production^16,21^. Studies have shown that using SBMA and MPC in dental materials can achieve long-lasting protein-repellent and antibacterial effects without compromising material properties relevant to clinical use^19,20^. The protein-repellent and anti-fouling properties of the two zwitterionic materials are generally attributed to its hydrophilicity and nature of the charge^16^. The oral cavity environment is characterized by the physical and mechanical changes due to functional and parafunctional activity^22^. Due to such dynamic mechanical stimulus luting cement is subjected to significant stresses. An effective luting cement material should therefore be capable to resist bacterial insult while offering adequate physical and mechanical properties^23^.

Taking the above into considerations, ZD can impart bacterial resistance properties to RMGIC. However, the chemically complex nature of the RMGIC may present variable changes in the luting cement properties following the addition of ZD. Furthermore, the optimal proportion of the ZD, which can be incorporated into the RMGIC without compromising the fundamental properties, has not been reported^24^. Hence, the purpose of the present study is to compare the effect of different concentrations of zwitterions on the physical properties of various commercially available RMGIC.

In this study, three representative RMGIC were compared (RelyX Luting 2, Nexus RMGI, GC FujiCEM 2) to identify the optimal acceptable concentration of zwitterions. In addition, the effect of two different ZD (SBMA and MPC) on the representative RMGIC groups was contrasted. The null hypotheses of this study were: (i) there would be no significant difference in physical properties in RMGIC with the addition of ZD in different concentrations, and (ii) the properties of RMGIC depend on the type of ZD.

## Materials and methods

### Materials

Three types of RMGIC that are commercially available in the market were used in this study. This study included self-cured system RelyX Luting 2 (3 M ESPE, St Paul, MN, USA), Nexus RMGI (Kerr Co, Orange, CA, USA), and GC FujiCEM 2 (GC Co, Tokyo, Japan). The selected materials are listed in Table 1.

**Table 1:** Compositions of resin-modified glass ionomer cement (RMGIC) used.

Group	Product	Manufacturer	Composition
RMC-I	RelyX Luting 2	3 M ESPE, St Paul, MN, USA	Paste A: Fluoroaluminosilicate glass, Proprietary reducing agent, HEMA, Water, Opacifying agent Paste B: Methacrylated polycarboxylic acid, Bis-GMA, HEMA, Water, Potassium persulfate, Zirconia silica filler
RMC-II	Nexus RMGI	Kerr, Orange, CA, USA	Paste A: HEMA, 2-hydroxy-1,3-propanediyl bismethacrylate, Ytterbium trifluoride, (1-methylethylidene) bis [4,1- phenyleneoxy(2-hydroxy-3,1-propanediyl)] bismethacrylate Paste B: None disclosed
RMC-III	GC FujiCEM 2	GC, Tokyo, Japan	Paste A: HEMA, Dimethacrylate component, UDMA, bis-EMA, Butylated hydroxytoluene Paste B: Polybasic carboxylic acid

Bis-GMA, bisphenol-A-di-glycidylmethacrylate; HEMA, hydroxyethyl methacrylate; TEGDMA, triethylene glycoldimethacrylate; UDMA, urethane-dimethacrylate monomer-1,6-bis-[methecryloyloxy-2-ethoxycarbonylamino], bis-EMA, Ethoxylated bisphenol-A dimethacrylate.

### Specimen preparation

Two types of ZD powder, sulfobetaine methacrylate (SBMA; Sigma-Aldrich, St. Louis, MO, USA) and 2-methacryloyloxyethyl phosphorylcholine (MPC; Sigma-Aldrich, St. Louis, MO, USA) were used to conduct this study. The RMGICs were supplied as two-pastes A and B when provided by the manufacturer. The ZD were mixed in paste A at different weight percentages (1, 3 wt.%) respectively. Then, components A and B were mixed homogeneously at a 1:1 ratio according to the manufacturer's instructions. The five groups were set as below:

1. Without ZD (Control)
2. Containing 1 wt.% SBMA in RMGIC (S1)
3. Containing 3 wt.% SBMA in RMGIC (S3)
4. Containing 3 wt.% MPC in RMGIC (M1)
5. Containing 3 wt.% MPC in RMGIC (M3)

### Experiment process and measurement metrics

#### Optical density

Bacterial analysis was carried out using Streptococcus mutans (*S. mutans*) (KCOM 3478) cultured in Brain Heart Infusion (BHI) broth (Difco, Sparks, MD, USA) with 2% sucrose added, and incubated at 37 °C for 18 h under aerobic condition. The samples for the bacterial experiment were manufactured in disc-shaped (10 mm × 2 mm) and sterilized in ethylene oxide gas. Then, 1 mL of the cultured bacterial suspension (1 × 10^8^ cells/mL) was placed into the specimens in a 24-well plate and incubated for 18 h. To remove the non-adherent bacteria, the samples were gently washed twice with distilled water. Afterward, the samples were sonicated (SH-2100; Saehan Ultrasonic, Seoul, Korea) for 5 min while immersed in distilled water for measuring the optical density (OD) values. The bacterial solution thus obtained after sonication was seeded (100 μL) in a 96-well plate and OD values (450 nm) were measured using a microplate reader (Epoch, BioTek Instruments, VT, USA)^25^.

#### Morphology

For microscopic examination, *S. mutans* attached to specimens were fixed with 2% paraformaldehyde–glutaraldehyde in 0.1 M PBS at 37 °C for at least 30 min^19^. Subsequently, specimens were post-fixed with 1% OsO_4_, dissolved in 0.1 M PBS for 2 h, dehydrated in ethanol, treated with isoamyl acetate, and dried to a critical point (LEICA EM CPD300; Leica, Wien, Austria). Thereafter, the discs were coated with Pt–ion (5 nm) using an ion coater (ACE600; Leica) and observed under scanning electron microscopy (FE-SEM; Merin, Carl Zeiss, Oberkochen, Germany) at 2 kV^26^.

#### Characterization of wettability

Wettability was determined by measuring the contact angle using a drop method (Smart Drop Lab, Femto Biomed Inc., Gyeonggi-do, Korea). Three disc-shaped samples (10 mm × 2 mm) were manufactured for this experiment. 5 μL droplet of distilled water was dropped on the sample surface, and the value was measured after 10 s^27^.

#### Film thickness

Film thickness was measured using two optically flat square glass plates with a contact surface area of 1600 mm^2^. All plates used in the experiment were standardized to the same area and thickness. The combined thickness of two glass plates (without the cement) was measured as measurement (A) using a micrometer (Mitutoyo 342–251, Sakado, Japan), accurate to 0.001 µm. The mixed cement (0.1 mL) was then dispensed directly between the two plates, and a vertical load (150 N) was applied for 180 s using the loading device according to ISO standard 4049: 2019^28^.

After the load, the thickness of the plates with the cement was measured in the same manner and recorded as measurement (B). The film thickness of RMGIC was calculated by subtracting the two values (B-A).

#### Flexural strength and elastic modulus

Flexural strength was performed according to the ISO standard 9917–2: 2010^29^. Within the material’s working time, the mixed cement was placed into the mold with dimensions of 25 mm (length) × 2 mm (width) × 2 mm (height) and pressed evenly on both sides using a flat microscope slide glass (76 mm × 26 mm × 1 mm; Paul Marienfeld GmbH, Bad Mergentheim, Germany). Subsequently, the specimen surface was polished and finished with a 400-grit abrasive paper^30^. Polished specimens were stored in a water bath (KMC-1205W, Vision Scientific Co., Ltd., Daejeon, Korea) at 37 °C and relative humidity of 100% for 24 h. The above procedure produced bar-shaped specimens (n = 5) to experiment with 3-point bending mechanical testing^31^.

Before the test, the height and width of the specimens were measured with 0.01 mm increments for precision using a digital micrometer (Mitutoyo digimatic caliper 500–181-30, Kawasaki, Japan). Each of the three parts were measured and the mean value was used.

The values in the flexural strength were loaded to fracture by using a universal testing machine (Instron 5942, Instron, Norwood, MA, USA) at a crosshead speed of 0.75 ± 0.25 mm/min. Flexural strength (σ) was calculated using Eq. (1), where F was the maximum load, L was the distance between the supports, *b* was the width of the specimen, and *h* was the height of the specimen.

1 $$\upsigma =\frac{3\mathrm{FL}}{2b{h}^{2}}$$

Elastic modulus (E) was calculated using Eq. (2), where F was the load at a point on the load–deflection curve and *d* was the deflection corresponding to load F.

2 $$\mathrm{E}=\frac{{\mathrm{FL}}^{3}}{4b{h}^{3}d}$$

#### Shear bonding strength

A total of ninety root-resected and cleaned bovine incisors obtained from the cattle slaughtered in accordance with the Korean animal slaughter regulations at permitted market were used in this study^32^. The bovine sacrifice had no relation with this study and was carried out for food processing. The prepared bovine crowns were embedded in a Teflon mold (20 mm diameter, 5 mm thickness) and filled with a self-cured acrylic resin (Polycoat EC-304, Aekyung Chemical, Chungnam, Korea). To expose the dentin surface, the resin-embedded samples were trimmed using a polishing machine (EcoMet 30, Buehler Ltd., Lake Bluff, IL, USA) in wet condition with 800 and 1200-grit carbide paper^33^ and stored in distilled water according to the ISO standard 29,022: 2013^34^.

Zirconia blocks (Zirtooth, HASS, Gangneung, Korea) were milled using a computer-aided milling machine (PM5‐All, Pistis, Incheon, Korea). The milled specimens were sintered based on the manufacturer's recommendation. Briefly, the samples were heat treated using the electric furnace (Lindberg, Asheville, NC) at 1000 Briefly, the samples were heat treated using (rate: 9 °C/min) followed by a stepped increment to 1500 °C at a heating rate of 3.5 °C/min and withheld for 2 hours^35^. Thereafter, the samples were cooled down to room temperature at a steady cooling rate of 9 °C/min. The zirconia ceramic surface was sandblasted for 10 s with a 50 µm alumina oxide particle at a 10 mm distance using 2.5 bar pressure and cleaned with 75% alcohol^36^.

**Figure 2:**
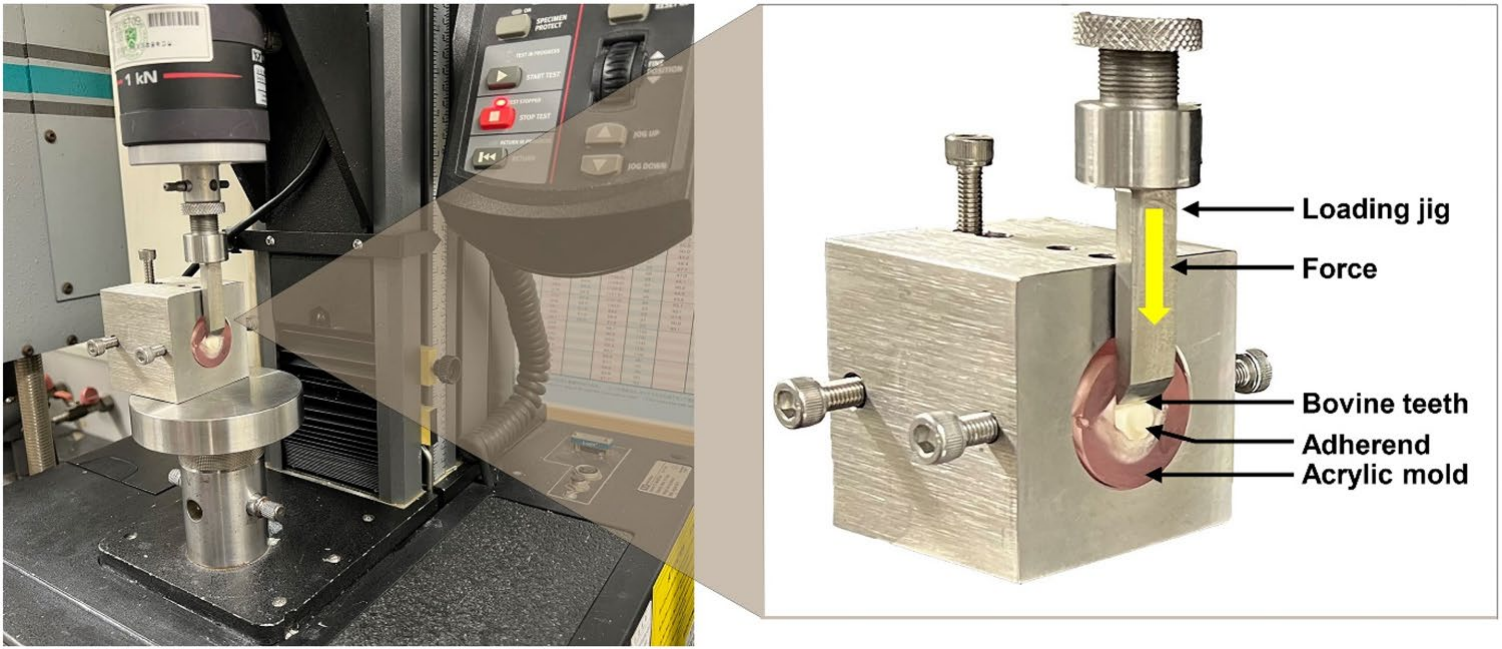
Schematic representation of the shear bond strength test.

The disc-shaped zirconia specimens were bonded with RMGIC to the bovine dentin surface under a constant load of 900 g^37^. Excess cement was removed with a micro-brush. Afterward, all specimens were stored in 37 °C water baths for 24 h^38^ and immersed in distilled water ready for the shear bonding strength test. The test was carried out using a universal testing machine (Instron 5942, Instron, Norwood, MA, USA) at a crosshead speed of 0.75 ± 0.25 mm/min until debonding (Fig. 2). Bond strength (σ) was calculated using Eq. (3), where F was the maximum load, in newtons (N), exerted on the specimen, π was the ratio of the circumference of a circle to its diameter (3.14), and *d* was the diameter of the zirconia.

3 $$\upsigma =\frac{\mathrm{F}}{\uppi {(d/2)}^{2}}$$

The failure mode was observed by debonding the samples using an optical microscope (Olympus SZ61, Tokyo, Japan) at 25 × magnification. Failure modes were classified into three types: adhesive failure (when less than 25% observable cement remained bonded on the dentin surface), cohesive failure (when failure occurred within the restoration, and more than 75% observable cement remained on dentin surface), and mixed failure (between 25 and 75% observable cement remained on dentin surface)^39^.

### Statistics

Test of distribution normality was performed with Shapiro–Wilk analysis. The statistical analyses of flexural strength, elastic modulus, and shear bonding strength were performed using the Kruskal–Wallis test followed by Dunn’s post-hoc test. The remaining results were analyzed by an one-way analysis of variance (ANOVA) and post-hoc Tukey’s test. All statistical analyses were conducted using a software program (IBM SPSS, version 23.0, IBM Korea Inc., Seoul, Korea) at a significance level of less than 0.05.

## Results

### Optical density (OD)

**Figure 3:**
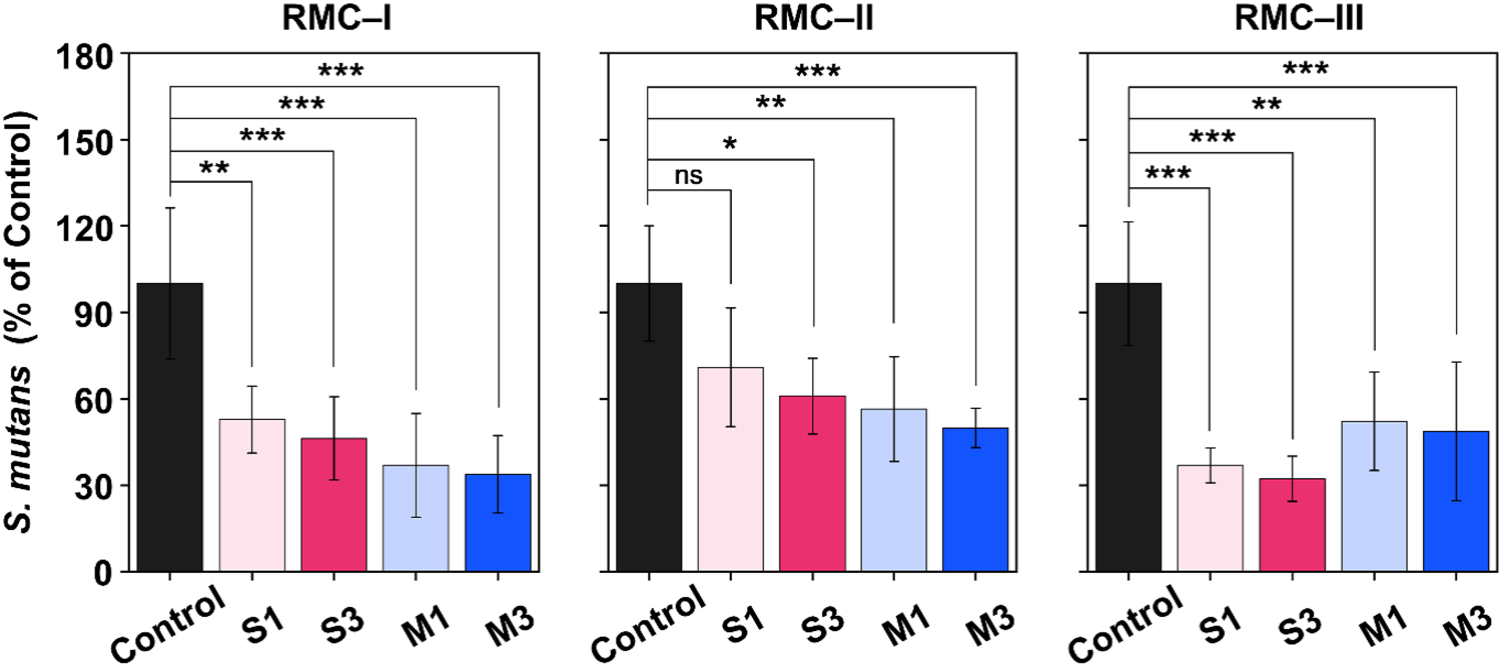
Optical density (OD_450_) readings derived from *S. mutans* attached on the surfaces of the RMC-I, II, and III. A varying number of asterisks indicate significant differences by post-hoc Tukey's test (* *P* < 0.05, ** *P* < 0.01, and *** *P* < 0.001).

Figure 3 illustrates the average OD values, where the control group OD value was fixed to 100% and the experimental groups calculated accordingly. The results from the three experimental groups showed that, except for S1 in the RMC-II group, the OD values of the experimental groups are significantly reduced compared to the control group (*P* < 0.001).

### Electron microscope observation

**Figure 4:**
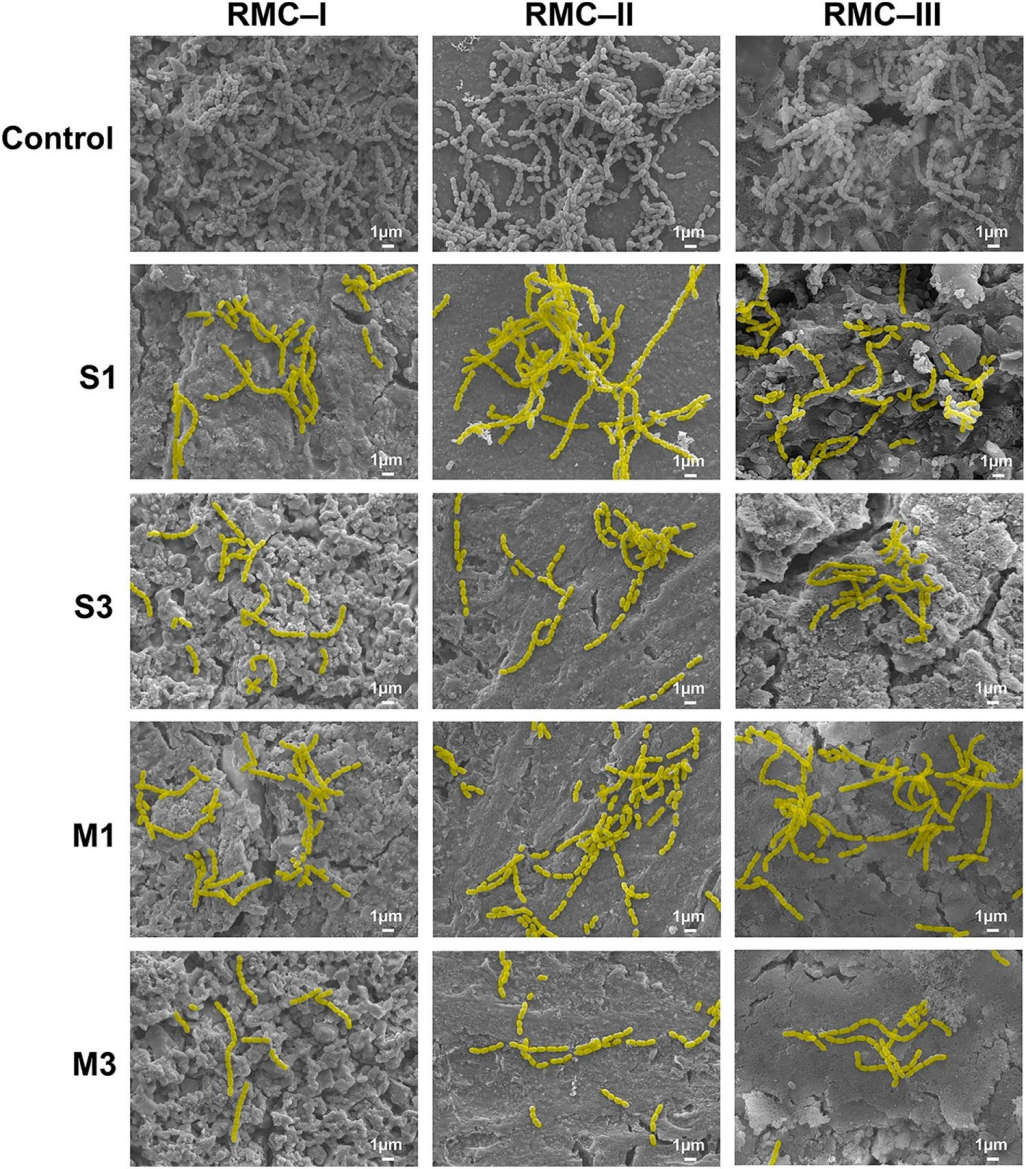
Representative scanning electron microscope images of *S. mutans* attached to the surfaces of control groups and experimental groups (magnification: 5000 ×). In the experiment groups, *S. mutans* attachment to the cement surface are pseudo-colored (yellow) for emphasis.

Figure 4 shows the SEM images of the *S. mutans* adhered to the RMGIC surface in the different groups. In RMC-I, *S. mutans* adhesion was notably less in all experimental groups when compared to the control group. Group M3 formed the lowest number of *S. mutans* adhesion. In addition, the group RMC-II showed fewer bacterial attachments at 3 wt.% ZD when compared to other samples. The images of group RMC-III images also showed similar observations to those of RMC-II.

### Contact angle

**Figure 5:**
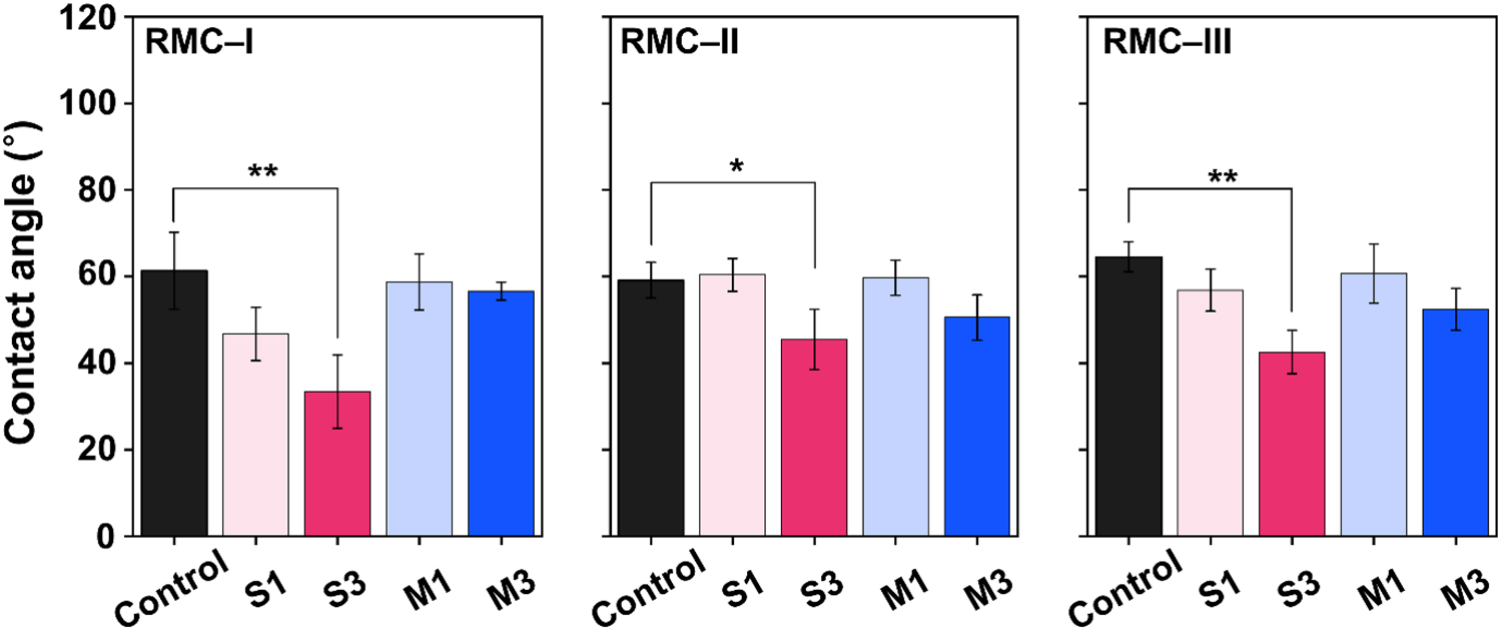
Surface wettability performance. A varying number of asterisks indicate significant differences by post-hoc Tukey's test (** *P* < 0.01, *** *P* < 0.001).

The results of the contact angle experiment are presented in Fig. 5. The RMC-I showed a decreasing trend in the S1 and S3 groups compared to the control group. The S3 group decreased significantly by 45.9% from the control group (*P* < 0.01). In RMC-II, only the S3 group revealed a significant difference compared to the control group (*P* < 0.05). Likewise, the RMC-III also showed a significant difference in the S3 group (*P* < 0.01). Overall, 3 wt.% concentration of ZD decreased the surface contact angle.

### Film thickness

**Figure 6:**
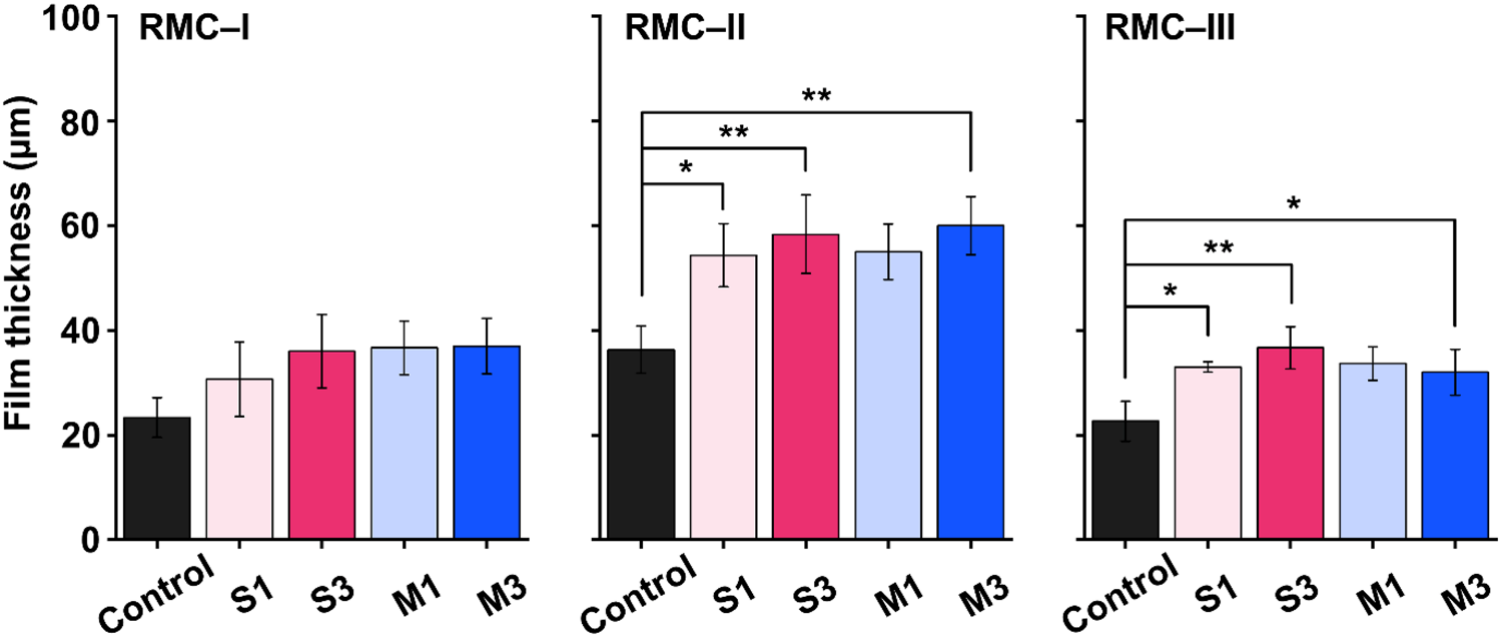
The film thickness of the control groups and experimental groups. A varying number of asterisks indicate significant differences by post-hoc Tukey's test (* *P* < 0.05, ** *P* < 0.01).

The measured film thickness is shown in Fig. 6. In the RMC-I, a statistically non-significant increase in film thickness was observed compared to the control (*P* > 0.05). Conversely, RMC-II exhibited significant differences in the 3 wt.% zwitterion groups (*P* < 0.01), however, M1 was least affected. The results from RMC-III also followed a similar trend. In summary, the film thickness of RMGIC was affected by ZD concentration in the order of RMC-II > RMC-III > RMC-I.

### Flexural strength and elastic modulus

**Figure 7:**
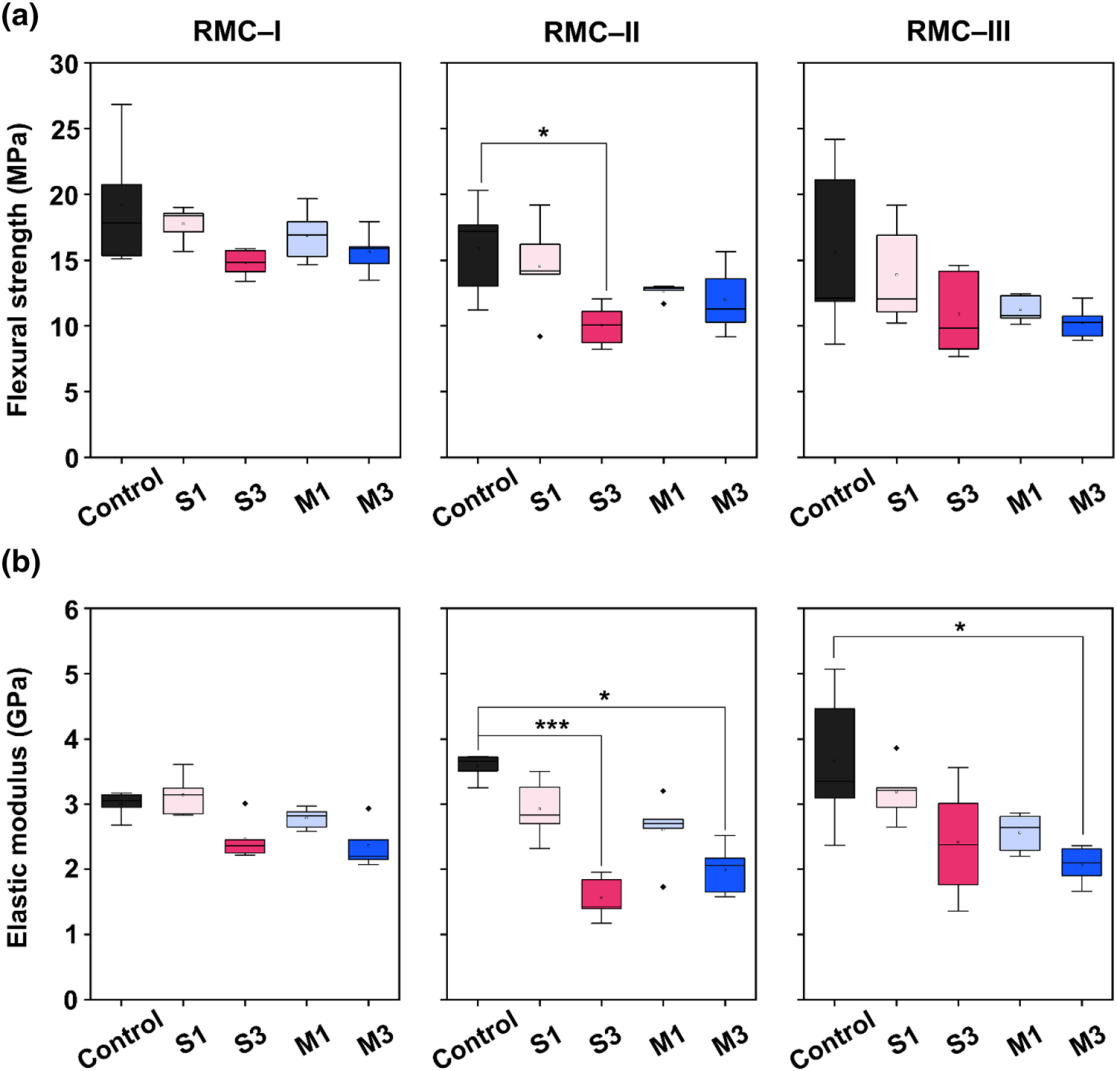
Flexural strength (**a**) and elastic modulus (**b**) of ZD-modified RMGICs. A varying number of asterisks indicate significant differences by post-hoc Dunn's test (* *P* < 0.05, *** *P* < 0.001). ZD; zwitterionic derivatives, RMGIC; resin-modified glass ionomer cement.

The flexural strength and elastic modulus are shown in Fig. 7a,b in box plot diagram. The plot shows the median shear bond strength and interquartile range values in all RMC groups. In RMC-I, the flexural strength decreased in all experimental groups, and S3 revealed the lowest value. Although the results were found to decrease, the differences were statistically insignificant (*P* > 0.05). In RMC-II, the flexural strength in S3 significantly decreased from the control (*P* < 0.05). In RMC-III, the decreasing trend was similar to that of RMC-I. The elastic modulus values in RMC-I revealed a decreasing trend in the S3 and M3 groups. However, there was no statistically significant difference (*P* > 0.05). S3 in the RMC-II showed the lowest values against the control (*P* < 0.001). The M3 group also showed a significant decrease (*P* < 0.05). In the RMC-III, M3 showed a significant decrease compared to the control group (*P* < 0.05).

### Shear bonding strength

**Figure 8:**
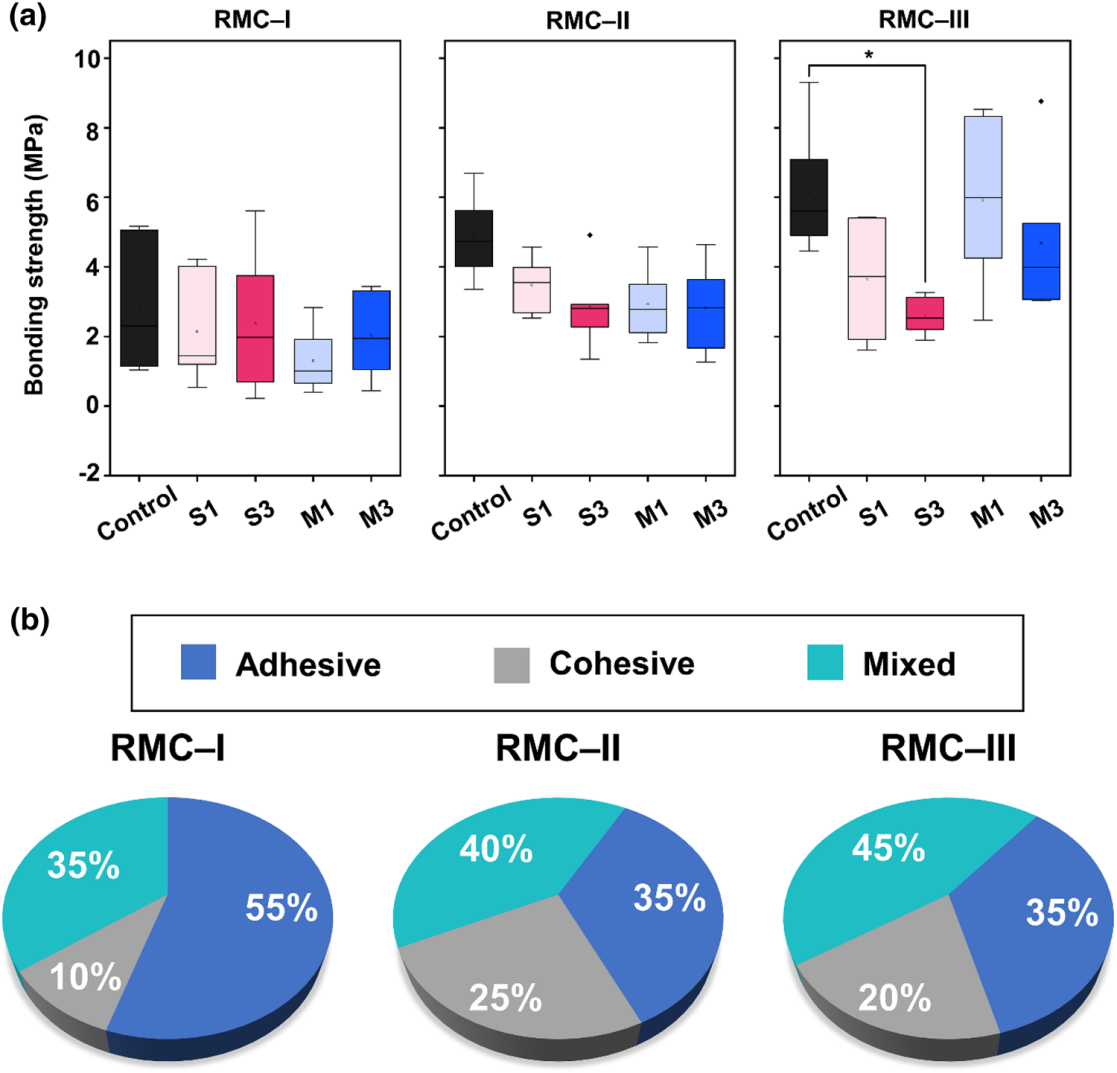
Shear bond strength (**a**) of ZD-modified RMGIC groups. (**b**) Percentage variation in the types of bonding failure observed per RMGIC group with ZD. The asterisk indicates a statistically significant difference for *P* < 0.05. ZD; zwitterionic derivatives, RMGIC; resin-modified glass ionomer cement.

The shear bonding strength results are presented in Fig. 8a. In the RMC-I, despite the reduced median value against the control, the differences were statistically insignificant in all groups (*P* > 0.05). In the RMC-II group, S3 presented a clear difference in shear bonding strength with a decreased median value of 41.4% when compared to control, but this was statistically insignificant (*P* > 0.05). Group S3 in RMC-III demonstrated a significantly lower median value of 57.9% against control (*P* < 0.05). In group M1 RMC-III, the difference between the median values was slight, but the interquartile range value appeared large. Figure 8b shows the percentage variation in the three bonding failure modes observed per the RMGIC group with ZD. The mode of failure differed slightly in each group, but all groups showed dominance in the adhesive and mixed failure.

## Discussion

According to this study’s results, the hypotheses are that adding different concentrations of ZD does not influence the properties of RMGI luting cement and that the properties of RMGI luting cement, depending on the types of ZD, could be rejected.

In this research, both 1 wt.% and 3 wt.% ZD in RMGICs significantly inhibited biofilm accumulation on different RMGICs, and the results were not statistically different between the two types of ZD groups. However, the capacity of RMGIC to inhibit biofilm does continue to increase as the concentration of ZD increases. The attachment of salivary proteins to enamel and dental material surfaces is a prerequisite for oral bacterial growth and biofilm formation^40^. Following the attachment of primary colonizers, cell-to-cell interaction pursues, leading to the colonization of cariogenic bacteria. Subsequently, a cariogenic *S.mutans*-rich biofilm is formed^41^. The ZD have a unique structure that can repel the adsorption of proteins by forming a large amount of free water around the functional group, thus inhibiting bacteria's adsorption to the material at the initial stage^16^. Moreover, the increase in hydrophilicity of the materials modified with ZD is also consistent with the previous view.

The film thickness of luting agents is a crucial characteristic and component of restorative dentistry. Cement with high viscosity will exhibit early settings before the end of the proper flow to reach the minimum film thickness^42^. During the bonding of the restoration, reducing the film thickness can also decrease the marginal discrepancies, which are essential for the complete seating and adaptation of the restoration. Furthermore, a thin film thickness reduces cement dissolution, intra-restoration plaque accumulation, and the progression of periodontal disease^43^. In the present study, RMC-I presented the most favorable thickness, with the incorporation of ZD being least affected by the ZD concentration. However, significant changes were observed in the RMC-II with a marked increase in thickness, emphasizing the impact of the inherent composition of the RMGIC and its interaction with ZD.

Determined materials parameters, such as flexural strength and elastic modulus, have typically been used to evaluate fracture-related material properties, such as fracture resistance, elasticity, and the marginal degradation of materials under stress^44^. Although flexural strength under constant loading might not accurately represent intraoral circumstances, these data are helpful for contrasting materials in laboratory settings^45^.

In RMC-I, the concentration of ZD did not express significant changes in flexural properties with S1, S3, M1, and M3. The findings were in contrast with the RMC-II and RMC-III results. The S3 and M3 specimens in RMC-II and RMC-III had dramatically reduced flexural properties compared with S1 and M1 specimens, and the changes were statistically significant. It may be related to chemical composition and content differences between different materials. According to a previous study, the flexural strength of RMC-II decreases dramatically between 1 and 24 h with 3 wt.% ZD having a greater impact on RMC-II^46^. Though the ZD modification affected the flexural properties of RMGIC, albeit to varying degrees, the cement still fulfilled the requirements set by the International Standard, ISO 9917–2^29^. The findings show that, rather than the type of ZD, the content of ZD present has a greater impact on the flexural properties of the RMGIC tested. This might be due to the increase in ZD content, which increases the water absorption of RMGIC. Water absorbed by the ZD spreads through RMGICs and mostly works as a plasticizer, lowering RMGIC's flexural strength and hardness. Additionally, water partially dissolved the cement component, changing the network of the RMGIC and slightly lowering its flexural strength and hardness^47^.

The SBS test is the most widely used test method because shear forces occur during the restoration, are simple to carry out, and have a low operational fault^48^. Typical values of RMGIC to dentin bond strength vary from 2.52 to 5.55 MPa and tend to show values near the upper end of these ranges^49^. Through the experimental results, adding ZDs decreased the bonding strength of RMC, and the bonding strength continued to decrease with an increase in ZDs content. It might be attributed to the hydrophilicity of ZD-containing luting cement causing osmotic pressure changes in the dentinal tubules by attracting the internal fluid. Such interaction can make the interphase layer between the dentin and luting cement unsuitable, consequently lowering the bonding strength. In addition, the film thickness and modulus of elasticity may also influence the bonding strength. The thicker film thickness interferes with the complete seating of the restoration, such as zirconia. It may have caused internal defects in the luting cement. Also, differences in the modulus of elasticity between cement and substrate (dentin or zirconia) may contribute to different outcomes of adhesive strength.

Failure mode analysis is an essential parameter for understanding the test results of adhesion strength between the two materials. Previous studies have reported a positive correlation between adhesive strength and failure mode^50^. There are three bonding failure modes, adhesive failure, cohesive failure, and mixed failure^48^. Higher bond strength has been reported to be associated with a higher proportion of cohesive and mixed failure modes, depending on study settings^51,52^. In agreement with these observations, the present study results had a notable proportion of mixed or cohesive failure modes, observed with RMC-II and RMC- III groups having high median shear bond strength. In contrast, adhesive failure mode was mainly observed in the RMC-I group in agreement with the shear bonding strength results (RMC-I < RMC-II < RMC- III), suggesting a reduced clinical applicability for RMC-I^53^.

In this study, the film thickness evaluation of ZD-modified RMGIC did not fully comply with ISO 9917–2^29^, and further refinement of the experimental method is needed to determine whether the film thickness of ZD-modified RMGIC meets the ISO standards. Additionally, this study was limited to an in vitro short-term setup only. Further investigations to elucidate the long-term effect of the ZD-modified RMGIC will significantly add to the present understanding. The future clinical trial will also help outline the efficacy and optimal protocol for using ZD-modified RMGIC as luting agents.

## Conclusion

It was concluded that incorporating both SBMA and MPC predictably improves the bacterial resistance of RMGIC. The addition of 1 wt.% ZD in RMGIC favorably enhanced bacterial resistance property without any tangible flexural and shear bond strength loss. An increase in ZD concentration beyond 1 wt.% can further improve bacterial resistance at the expense of the physical and mechanical properties of RMGIC.

## Data Availability

The data that support the findings of this study are available from the corresponding author upon reasonable request.
